# P-1570. Plasma IL-6 level predict the risk of in-hospital mortality in HIV-associated Pneumocystis Pneumonia

**DOI:** 10.1093/ofid/ofaf695.1750

**Published:** 2026-01-11

**Authors:** Huan Xia, Ping Ma

**Affiliations:** Tianjin Second People's Hospital, Tianjin, Tianjin, China; Tianjin Second People's Hospital, Tianjin, Tianjin, China

## Abstract

**Background:**

To determine plasma immune-inflammatory biomarkers that may predict in-hospital mortality in HIV-infected individuals diagnosed with pneumocystis *jirovecii* pneumonia (PCP).

**Table 1:**
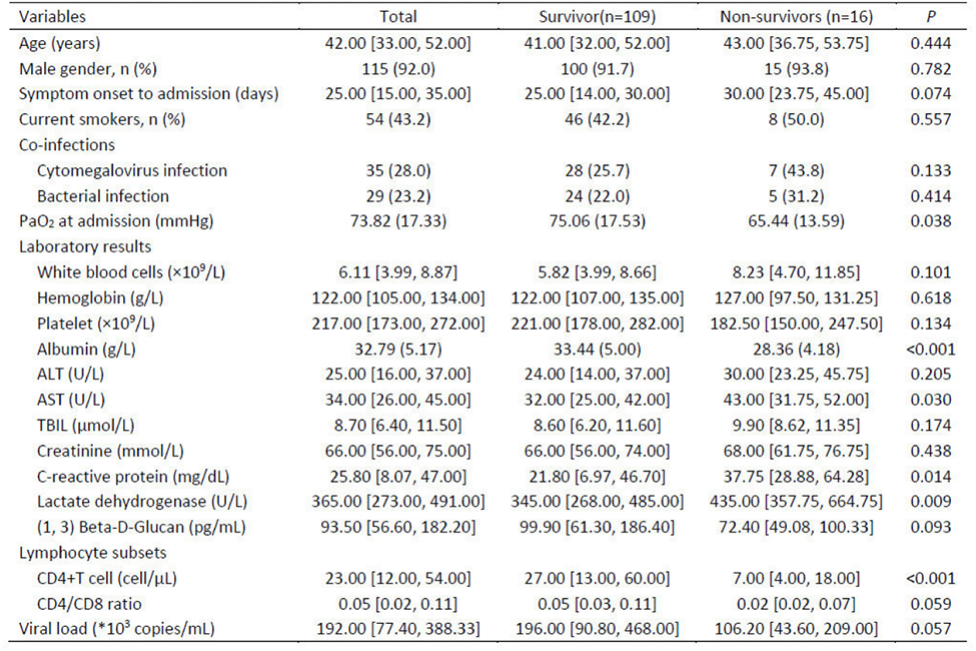
Characteristics of 125 HIV-infected patients with Pneumocystis pneumonia based on survival outcomes

**Figure 1:**
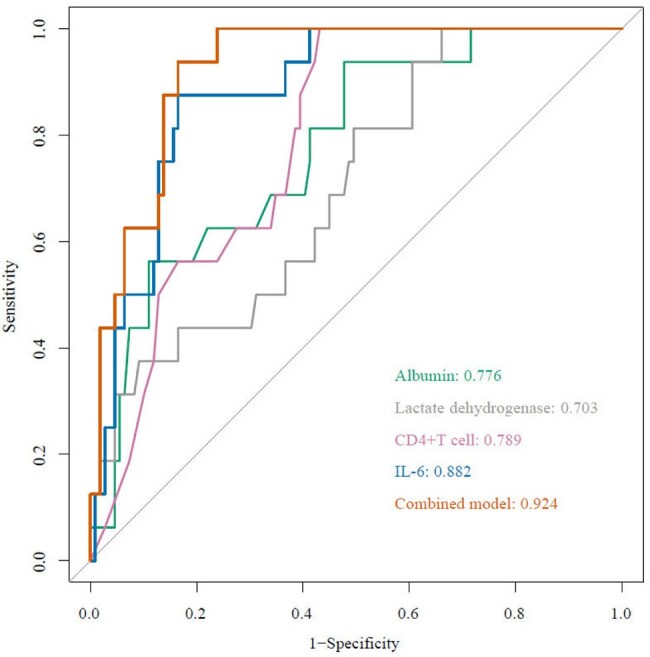
Receiver operating characteristic (ROC) curves for in-hospital mortality. IL-6 interleukine-6. The combined ROC curve represents the combination of IL-6 and other markers used to predict in-hospital mortality.

**Methods:**

This study prospectively included 125 HIV-infected patients with PCP. Biomarkers involving clinical variables and 8 pre-selected plasma inflammatory cytokines (IL-2, IL-4, IL-6, IL-10, IL-2, IL-4, IL-6, IL-10, IL-12, IL-17, TNF-α, and IFN-*γ*) were evaluated at time of admission. Multivariate logistic regression analysis was used to identify factors substantially associated with in-hospital mortality. The predictive value of the biomarkers for in-hospital mortality was assessed using the ROC curve.

**Results:**

Our results show a hospital mortality rate of 12.8% (16/125). When compared to surviving AIDS PCP patients, non-survivors had substantially higher levels of C-reactive protein, IL-6, aspartate aminotransferase, and lactate dehydrogenase and lower levels of albumin, PO_2_, and CD4 count. We found a significant association between increased IL-6 levels and hospital mortality using multivariable logistic regression analysis (adjusted odd ratio, 1.006; 95% CI, 1.002-1.012; P = 0.012). The plasma IL-6 levels had a maximum area under the Area Under Curve (AUC) (0.883; 95%CI, 0.812-0.953), compared to CD4+ T cell (AUC, 0.789; 95%CI, 0.697-0.881), ALB (AUC, 0.776; 95%CI, 0.661-0.892), and LDH (AUC, 0.703; 95%CI, 0.573-0.832).

**Conclusion:**

A high level of plasma IL-6 has been associated to an elevated probability of in-hospital mortality.

**Disclosures:**

All Authors: No reported disclosures

